# Health and economic burden of sugar-sweetened beverages consumption in Brazil

**DOI:** 10.1590/0102-311XEN249422

**Published:** 2023-12-22

**Authors:** Lucas Perelli, Andrea Alcaraz, Cid Manso de Mello Vianna, Natalia Espinola, Federico Rodriguez Cairoli, Ariel Bardach, Alfredo Palacios, Dario Balan, Paula Johns, Federico Augustovski, Andrés Pichón-Rivière

**Affiliations:** 1 Instituto de Efectividad Clínica y Sanitaria, Buenos Aires, Argentina.; 2 Instituto de Medicina Social, Universidade do Estado do Rio de Janeiro, Rio de Janeiro, Brasil.; 3 Consejo Nacional de Investigaciones Científicas y Técnicas, Buenos Aires, Argentina.; 4 Centre for Health Economics, University of York, York, U.K.; 5 ACT Promoção da Saúde, Rio de Janeiro, Brasil.

**Keywords:** Sugar Sweetened Beverages, Illness Burden, Noncommunicable Diseases, Bebidas Azucaradas, Costo de Enfermedad, Enfermedades No Transmisibles, Bebidas Adoçadas com Açúcar, Carga da Doença, Doenças Não Transmissíveis

## Abstract

Sugar-sweetened beverages (SSBs) are a major source of added sugar and are associated with noncommunicable diseases (NCDs) such as obesity and diabetes. This study assessed the impact of SSBs consumption on disease burden in Brazil, including deaths, disability-adjusted life years (DALYs), and healthcare costs. A 3-stage methodology was used to assess the direct effects of SSBs on diabetes, cardiovascular diseases, and body mass index (BMI), along with the influence of BMI on disease incidence. These assessments were then used to estimate the economic and health burden using population-attributable factors. Results showed that 2.7% and 11% of adult and children overweight/obesity cases were attributable to SSBs, respectively. SSBs consumption in Brazil led to 1,814,486 cases, 12,942 deaths, 362,088 DALYs, and USD 2,915.91 million in medical costs related to diabetes, cardiovascular diseases, oncological diseases, and other NCDs. Urgent implementation of public policies is crucial to address the consumption of SSBs, recognized as a key risk factor for NCDs.

## Resumen

Las bebidas azucaradas (BA) tienen una gran fuente de azúcar añadido y están asociadas con enfermedades no transmisibles (ENT), como la obesidad y la diabetes. Este estudio evaluó el impacto del consumo de las BA en la carga de enfermedad en Brasil, incluidas las muertes, los años de vida ajustados por discapacidad (AVAD) y los costos con la salud. Con el uso de una metodología de tres etapas, se evaluaron los efectos directos de las BA sobre la diabetes, las enfermedades cardiovasculares y el índice de masa corporal (IMC), la influencia del IMC en la incidencia de la enfermedad, y se estimó la carga económica y de salud utilizando los factores atribuibles a la población. Los resultados mostraron que el 2,7% de los casos de sobrepeso/obesidad en adultos y del 11% en niños fueron atribuibles a las BA. El consumo de las BA en Brasil generó 1.814.486 casos, 12.942 muertes, 362.088 AVAD y USD 2.915,91 millones en costos médicos relacionados con diabetes, enfermedades cardiovasculares, enfermedades oncológicas y otras ENT. Es necesario implementar políticas públicas para tratar el consumo de las BA, reconocido este como un factor de riesgo clave para las ENT.

## Resumo

As bebidas açucaradas (BAs) são uma grande fonte de açúcar adicionado e estão associadas a doenças não transmissíveis (DNTs), como obesidade e diabetes. Este estudo avaliou o impacto do consumo de BAs sobre a carga de doenças no Brasil, incluindo óbitos, anos de vida ajustados por incapacidade (AVPIs) e custos de saúde. Usando uma metodologia de três estágios, examinamos os efeitos diretos das BAs sobre diabetes, doenças cardiovasculares e índice de massa corporal (IMC), a influência do IMC na incidência de doenças e estimamos o carga econômica e de saúde usando fatores atribuíveis à população. Os resultados mostraram que 2,7% dos casos de sobrepeso/obesidade em adultos e 11% em crianças foram atribuíveis a BAs. O consumo de BAs no Brasil levou a 1.814.486 casos, 12.942 mortes, 362.088 AVPIs e USD 2.915,91 milhões em custos médicos relacionados a diabetes, doenças cardiovasculares, doenças oncológicas e outras DNT. A implementação urgente de políticas públicas é crucial para enfrentar o consumo de BAs, reconhecido como um fator de risco fundamental para as DNT.

## Introduction

Noncommunicable diseases (NCDs) are the product of a combination of genetic and environmental risk factors influenced by physiology and behavior ^1^. NCDs are responsible for most deaths worldwide yearly, and the disease burden is much more significant in low- and middle-income countries, with more than 80% of premature deaths from NCDs occurring in these countries. Overweight and obesity are two of the most important determinants of the burden of disease and death currently attributable to NCDs, with high body mass index (BMI) responsible for more than 4 million deaths and 120 million disability-adjusted life years (DALYs) worldwide in 2019 ^2^. Even though obesity occurs worldwide, its incidence has rapidly increased in low- and middle-income countries. In Latin America, the rate of obesity is growing faster than anywhere else in the world ^3^. In Brazil, 96 million people are affected by overweight, and 29.5% and 21.8% of Brazilian women and men live with obesity, respectively ^4^. In recent decades, an increase in NCDs has been noted in Brazil, closely related to the rise in obesity ^5^. Moreover, this problem is not only relevant in the adult population since, in Brazil, in 2019, it was found that 7% and 3% of children up to 5 years old were suffering from overweight and obesity, respectively; moreover, regarding adolescents aged 15 to 17 years, it was found that 19.4% and 6.7% were afflicted with overweight and obesity, respectively ^4^ ^,^ ^6^. People living with obesity are at risk of developing type 2 diabetes mellitus, cardio and cerebrovascular diseases, renal disorders, osteoarthritis, and several types of cancer, such as esophageal, uterine, and colon cancers ^7^.

High consumption of sugars has been associated with an increased risk of developing NCDs, including obesity and dental caries ^8^ ^,^ ^9^. Sugar-sweetened beverages (SSBs) are high in calories, low in nutritional value, and the diet’s first source of added sugar. Its consumption varies considerably according to sociodemographic characteristics, being higher in young people, in the male sex, and in America in relation to other regions. SSBs promote weight gain and increase the risk of other cardiovascular and metabolic disorders, such as type 2 diabetes mellitus. In addition, reducing their consumption promotes weight loss and thus reduces the risk of obesity-related diseases, such as cardiovascular disease (CVD), cancer, musculoskeletal disorders, asthma, depression, social isolation, dental caries, among others. It should be noted that these diseases attributable to the consumption of SSBs imply a great cost for health systems and society in general; therefore, this enormous disease and economic burden is a significant barrier to development.

In this context, it is essential for policy makers to have data that allow estimating the burden of disease attributable to SSBs consumption both in terms of health consequences and associated costs. Although extensive literature exist on the burden associated with this health detrimental consumption, to the best of our knowledge, no regional estimate addressing the economic burden attributable to SSBs consumption exist ^10^ ^,^ ^11^. This study is part of a collaborative effort between researchers, policymakers, and scientists from different universities, research centers, and government institutions in Argentina (Institute for Clinical Effectiveness and Health Policy), Brazil (ACT Health Promotion and the State University of Rio de Janeiro), El Salvador (Ministry of Health), and Trinidad and Tobago (University of the West Indies). This study aimed to estimate the burden of disease attributable to SSBs consumption in terms of deaths, events, DALYs, and direct medical costs to the Brazilian health system.

## Methods

### Model structure

**Figure 1:**
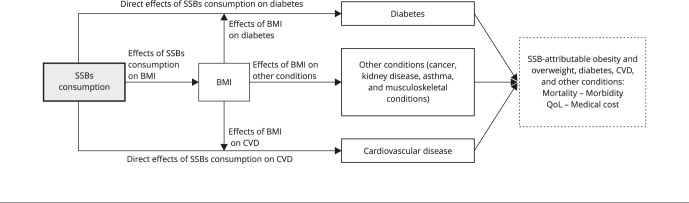
Model for causal framework and main outcomes.

BMI: body mass index; CVD: cardiovascular disease; QoL: quality of life; SSBs: sugar-sweetened beverages. Note: CVD - include stroke ischemic, intracerebral hemorrhage, subarachnoid hemorrhage, atrial fibrillation and flutter, ischemic heart disease, and hypertensive heart disease; Other conditions include oncologic diseases (cancers of the colon and rectum, esophagus, gallbladder, biliary tract, kidney, and uterus) and non-oncologic diseases (dementia, asthma, chronic kidney disease, gallbladder and biliary diseases, low back pain, and osteoarthritis).

The assessment of SSBs’ economic and health burden was conducted using a comparative risk assessment framework. A model with two main mechanisms was used to assess the yearly burden attributable to SSBs consumption. In the first path, SSBs consumption directly affects BMI, which contributes to the health and economic effects of overweight, obesity, and related diseases. In the second path, SSBs consumption directly affects type 2 diabetes mellitus and CVD (Figure 1). The initial model structure and the selection of outcomes were based on a systematic review of models to assess the burden of diseases attributable to SSBs consumption in international scientific literature ^12^. In addition, a policy dialogue was held, attended by experts and decision-makers from Argentina, Brazil, El Salvador, and Trinidad and Tobago ^13^. As a result of these activities, the model was developed to meet the information needs of decision-makers to control SSBs throughout the region. For children and adolescents, the model estimates only the impact of SSBs consumption on the prevalence of overweight and obesity.

To calculate the impact of SSBs consumption on each health event, the population attributable fraction (PAF) was used. By reducing exposure to a risk factor (SSBs consumption) to a minimal theoretical risk exposure scenario (zero consumption), the PAF was used to estimate a proportional reduction in disease and mortality in the population. By applying the population impact factor attributed to a risk factor to the total number of deaths, cases, or costs, the number of deaths, cases and costs were quantified. We used the PAF equation as follows:

$$PAF=\frac{\int_{i=1}^{max}P_{i}{RR}_{i}-\int_{i=1}^{max}P_{i}^{'}{RR}_{i}}{\int_{i=1}^{max}P_{i}{RR}_{i}}$$

where *i* = SSBs consumption level; *P* = current distribution in the age and sex stratum; *P’* = alternative distribution (zero consumption); *RR* = relative risk of mortality/cases/costs at SSBs consumption level *i*; and *max* = maximum exposure level.

The methodology employed in this study consisted of a three-stage process to assess the impact of SSBs on both health outcomes and economic burdens. In the first stage, the direct effect of variations in SSBs consumption were quantified on the incidence of type 2 diabetes mellitus, CVD, and changes in the BMI. Subsequently, the influence of BMI was estimated on the occurrence of the related diseases considered. The diseases linked to an increase in BMI considered for this model were asthma, atrial fibrillation and flutter, chronic kidney disease, colon and rectal cancer, dementia, esophageal cancer, gallbladder and bile duct cancer and kidney cancer, gallbladder and bile duct disease, hypertensive heart disease, intracerebral hemorrhage, low back pain, osteoarthritis, and subarachnoid hemorrhage. Finally, the population attributable factor was determined to estimate the economic and health burden associated with SSBs consumption. This model estimates the number of cases, deaths, DALYs, and healthcare costs associated with SSBs consumption in Brazil in 2020. The sensitivity analyses were performed using Monte Carlo simulation, which examined the impact of uncertainty regarding SSBs consumption and the relative risk (RR) linking SSBs to BMI, type 2 diabetes mellitus, and CVD. For each sex and age group, 1,000 iterations were performed using SSBs consumption distribution (mean and standard error), and relative risks were calculated using normal distributions and standard deviations. The mean and 95% confidence interval (95%CI) were reported. Moreover, this analysis was performed using Stata, version 14.04 (https://www.stata.com), and Visual Basic Excel programs (https://products.office.com/).

### Epidemiological data

Concerning the epidemiological parameters, the following sources of data were prioritized: (i) Brazilian sources, when available, (ii) Latin American sources when local information was not available, (iii) international sources, and (iv) estimates from the research group when data was not considered transferable from other sources.

The SSBs were defined as sports and energy drinks, sugar-sweetened sodas, sugar-sweetened fruit juices, and sugar-sweetened and flavored waters. The SSBs category did not include products containing sugar such as dairy products, coffee, *mate*, or tea. To estimate the consumption of SSBs in Brazil, data from the 2017-2018 *Household Budgets Survey* (POF) were used according to sex and the age strata considered ^14^. One serving was defined as the intake of 240mL of any SSB.

Regarding the impact of SSBs consumption on BMI, a 0.10kg/m^2^ (95%CI: 0.05-0.15) increase in BMI of subjects with basal BMI < 25kg/m^2^, and a 0.23 (95%CI: 0.14-0.32) increase in BMI of subjects with basal BMI ≥ 25kg/m^2^, was inputted per each serving of SSB consumed per day in adults ^15^. Moreover, BMI reductions of ~ 0.57kg/m^2^ were calculated for a 1.7 servings reduction in SSBs consumption of children ^16^.

Overweight and obesity prevalence data were obtained from the latest national risk factor survey conducted (2018), data were gathered on the demographic structure, incidence, prevalence, and mortality of each disease, stratified by sex and age ^14^.

The DisMod II software (https://www.epigear.com/index_files/dismod_ii.html) was used to model missing information regarding incidence, prevalence, or case-fatality rates for each disease ^17^. RR that quantifies the direct association between SSBs consumption and type 2 diabetes mellitus incidence and CVD incidence/mortality were identified. For type 2 diabetes mellitus, a 1.37 RR (95%CI: 1.15-1.63) increase was calculated on incidence per serving per day ^18^. For CVD, a 1.08 (95%CI: 1.04-1.13) and 1.08 (95%CI: 1.02-1.14) RR per serving per day were considered for incidence and mortality, respectively ^19^.

The remaining diseases included (cancers of the esophagus, colon, rectum, uterus, kidney, gallbladder, and biliary tract; osteoarthritis; low back pain; asthma; dementia; chronic kidney disease; and biliary tract and gallbladder diseases) were modelled by an increase in the BMI (indirect pathway). The respective RR of developing each disease from different basal BMI measures were obtained from the Institute of Health Metrics and Evaluation (IHME) ^7^.

### Direct medical costs

The direct medical costs of diagnosis, treatment, and follow-up were estimated for each disease included in the model. The direct medical costs represent the cost of the public health system. A micro costing approach was used to estimate the direct medical cost of the following conditions: type 2 diabetes mellitus, overweight, obesity, acute myocardial infarction, heart failure, renal failure (with and without dialysis), and stroke. Estimates were developed primarily based on consultation with clinical experts and, when possible, also based on international clinical guidelines ^20^ ^,^ ^21^. The costs of incident diseases were considered and, for chronic pathologies, a differentiation was made between the costs of the first year and subsequent years. The direct medical costs were estimated in local currency units (Brazilian Real) and then converted to US Dollars using the exchange rates of 2020, published by the Brazilian Central Bank ^22^.

## Results

The model’s main epidemiological and economic parameters are detailed in Tables 1 and 2.

**Table 1:** Main epidemiological parameters.

Gender/Age group (years)	Population (millions) *	SSBs consumption (servings mean) **	Overweight (%) **	Obesity grade 1 (%) **	Obesity grade 2 (%) **	Obesity grade 3 (%) **
Adults						
Female						
18-44	45.0	0.8	27.57	10.00	2.62	1.10
45-64	22.8	0.5	37.26	16.04	4.67	1.76
> 65	10.0	0.3	37.61	15.87	4.08	1.55
Subtotal	77.8	0.5	33.62	13.68	3.75	1.41
Male						
18-44	44.0	1.08	38.44	14.19	3.54	1.44
45-64	21.2	0.6	45.69	16.85	3.87	1.17
> 65	7.0	0.3	42.00	13.54	2.23	0.63
Subtotal	72.2	0.6	41.70	14,60	3.36	1.15
Both						
18-44	89.0	0.93	33.03	11.58	3.08	1.20
45-64	44.0	0.5	41.23	16.39	4.19	1.49
> 65	17.0	0.3	39.61	14.18	3.41	1.09
Subtotal	150.0	0.6	37.70	14.14	3.55	1.28
Children and teenagers						
Female						
0-4	7.2	0.5	7.60	7.40		
5-17	19.8	1.17	6.58	2.03		
Subtotal	27.0	1.0	7.10	4.70		
Male						
0-4	7.5	0.4	7.60	8.30		
5-17	20.6	1.29	8.37	3.60		
Subtotal	28.1	1.1	8.00	4.30		
Both						
0-4	14.7	0.4	7.60	7.80		
5-17	40.4	1.21	7.48	2.81		
Subtotal	55.1	1.0	7.50	6.40		

SSB: sugar-sweetened beverage.

* Source: Brazilian Institute of Geography and Statistics ^31^;

** Source: Brazilian Institute of Geography and Statistics ^32^.

**Table 2:** Economic and other epidemiological parameters of the model.

Diseases	Incidence rate *	Prevalence rate *	Mortality rate *	Incident event costs (USD)	Costs of 2+ year per prevalent event (USD)
Asthma	1,021.77	4,665.80	1.16	1,453.80	
Atrial fibrillation and flutter	46.09	640.80	4.840	960.04	
Chronic kidney diseases	268.62	7,337.15	16.10	1,504.69	
Colon and rectum cancer	16.30	69.600	10.28	14,699.91	1,918.04
Dementia	94.50	603.70	35.50	1,996.65	
Type 2 diabetes mellitus	195.70	3,834.20	20.82	1,635.20	
Esophageal cancer	4.60	6.57	4.84	22,403.94	15,174.22
Gallbladder and biliary diseases	69.30	433.60	2.92	271.46	
Gallbladder and biliary tract cancer	2.20	1.60	2.34	18,136.41	12,415.07
Hypertensive heart disease	46.90	211.50	10.60	1,122.53	
Intracerebral hemorrhage	33.90	189.70	27.90	1,683.61	
Ischemic heart disease	78.80	1,563.70	80.00	6,210.62	345.45
Ischemic stroke	67.21	856.20	22.60	1,683.61	
Kidney cancer	44,717.00	28.900	1.77	19,204.09	13,450.09
Low back pain	4,528.15	11,018.36	NA	16.55	
Osteoarthritis	160.48	3,142.85	NA	372.66	
Subarachnoid hemorrhage	16.44	144.18	6.06	5,528.82	
Uterine cancer	3.59	25.78	1.13	9,094.23	898.76

NA: not available.

Note: country economic data - gross domestic product (GDP) per capita: USD 8,717; percentage of GDP spending on health: 9.50%; USD exchange rate: 3.94.

* Per 100,000 inhabitants.

The average consumption of sugar-sweetened beverages in adults over 18 years of age was 52 liters per year per person, corresponding to 0.6 servings per day, whereas children and adolescents were found to drink 87,6 liters per year per person (1 serving per day). Marked differences were observed by sex and age groups (Table 1).

Table 3 details the events, deaths, and direct medical costs attributable to SSBs consumption for each disease for both males and females. This number of cases could be avoided if no SSBs were consumed.

**Table 3:** Attributable burden due to sugar-sweetened beverages (SSB) consumption in Brazil.

Condition	Women		Men		Both	
	Total attributable (95%CI)	%	Total attributable (95%CI)	%	Total attributable (95%CI)	%
Overweight and obesity (< 18 years)						
Events	331,630 (172,447-461,495)	11.00	391,009 (203,324-543,502)	7.00	722,639 (381,499-1,006,604)	11.00
Direct medical costs (USD million)	13.33 (7.2-18.3)	14.00	8.42 (4.54-11.6)	10.00	21.74 (11.7-30)	12.00
Overweight and obesity (adults)						
Events	829,703 (613,980-1,028,204)	2.20	1,389,465 (102,824-1,806,304)	3.20	2,219,168 (1,647,187-2,907,689)	2.70
Direct medical costs (USD million)	37.24 (27.7-54.7)	3.00	75.3 (56-111)	6.70	112.54 (83.8-165.7)	4.80
Type 2 diabetes mellitus						
Deaths	2,672 (1,054-4,546)	10.90	2,591 (1,117-4,195)	13.60	5,225 (2,181-8,511)	12.07
Events	688,369 (295,545-1,041,745)	15.00	727,013 (323,894-1,089,068)	19.00	1,415,383 (624,131-2,129,006)	17.00
Direct medical costs (USD million)	1,126 (506.7-1,722.78)	15.20	1,189 (503-1,819)	18.00	2,265 (1,021-3,482)	16.30
Heart diseases *						
Deaths	1,444 (605-2,441)	2.60	2,220 (1,018-3,581)	3.35	3,664 (1,654-5,910)	3.02
Events	51,967 (23,157-83,174)	2.93	87,365 (41,486-138,275)	3.76	139,332 (65,165-217,649)	3.40
Direct medical costs (USD million)	81 (37.6-127)	2.90	132 (61.3-206)	3.07	213 (99-334)	3.30
Cerebrovascular disease						
Deaths	1,533 (659-2,574)	2.65	1,945 (893-3,107)	3.37	3,479 (1,568-5,593)	3.00
Events	42,880 (19,646-67,369)	3.20	49,743 (23,776-78,421)	3.96	92,938 (43,293-143,770)	3.58
Direct medical costs (USD million)	104 (48.1-160)	3.35	110 (50.8-169.6)	4.00	214 (99-330)	3.60
Other conditions **						
Deaths	260 (161-394)	0.22	313 (194-474)	0.35	573 (354-868)	0.27
Events	96,498 (60,996-149,951)	0.56	82,753 (52,308-128,592)	0.46	179,251 (113,304-278,544)	0.53
Direct medical costs (USD million)	88 (55.5-133.5)	0.30	87 (54-132)	0.04	175 (110-265)	0.33
Total						
Deaths	5,910 (3,247-8,940)	2.60	7,032 (4,310-10,175)	3.36	12,942 (8,070-18,500)	2.96
Events	857,242 (203,122-1,462,143)	2.02	957,243 (259,976-1,576,838)	2.68	1,814,485 (463,098-3,038,981)	2.32
Direct medical costs (USD million)	1,399 (801-1,999.1)	3.80	1,517 (869.42-5,813.48)	5.30	2,865 (1,642-4,094)	4.50

95%CI: 95% confidence interval.

Note: USD exchange rate source: World Bank ^33^.

* Includes: atrial fibrillation, ischemic heart disease and hypertensive heart disease;

** Includes: cancer, oosteoarthritis, asthma, chronic kidney disease, dementia, and biliary diseases.

### Overweight and obesity

**Figure 2:**
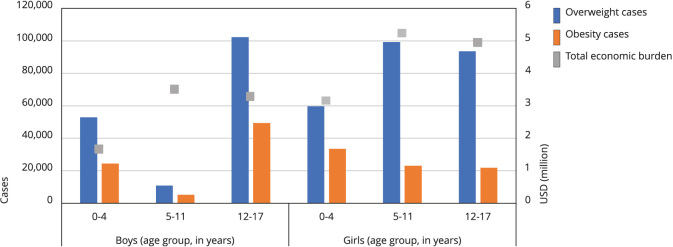
Burden of overweight and obesity attributable in children by sex and age groups in Brazil.

**Figure 3:**
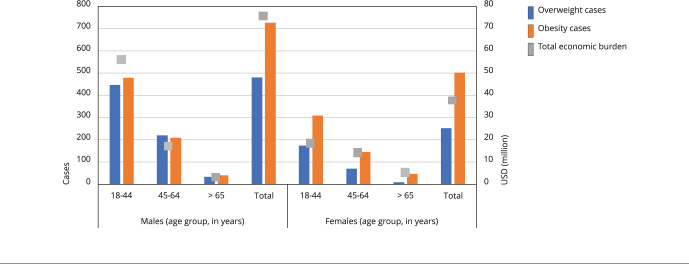
Burden of diseases attributable to overweight and obesity in adults by sex and age groups in Brazil.

A total of 722,639 cases of obesity and overweight in children and adolescents are attributable to SSBs consumption, representing 11% of the total cases of obesity and overweight in this population. In the adult population over 18 years of age, a total of 2,219.16 cases of obesity or overweight can be attributed, representing 2.7% of the total number of cases of both conditions. The direct medical costs for treating these cases of obesity and overweight attributable to SSBs consumption totaled USD 21.74 million per year in children and adolescents and USD 112.54 million per year in the adult population. Figures 2 and 3 illustrate the burden of overweight and obesity attributable in adults and children by sex and age groups in Brazil.

### Diabetes, cardiovascular disease, and other chronic diseases

The model estimated that, for the adult population, 1,814,486 cases and 12,942 deaths from type 2 diabetes mellitus, CVD, oncological diseases, and other chronic NCDs can be attributed to the consumption of SSBs in Brazil. These numbers could be avoided in a hypothetical scenario without consumption of SSBs, using population data for the year 2020. This disease burden represents 362,088 DALYs and USD 2,915.91 million in direct medical costs. The proportion of cases was 10.9% higher in males than in females, while a similar difference was observed in attributable deaths (11.8%, with 5,951 attributable deaths in females and 7,069 deaths in males) (Table 3).

The largest number of cases, deaths, and attributable DALYs corresponded to type 2 diabetes mellitus. An estimation of 1,415,383 cases and 5,292 deaths due to complications secondary to type 2 diabetes mellitus accounted for 16.63% of all cases and 12.07% of deaths due to type 2 diabetes mellitus in Brazil by 2020. These values represent 190,976 DALYs lost and an annual cost of USD 2,314.44 million for its treatment, which represents the 16.73% of the total direct costs attributable to the treatment of diabetes mellitus at the national level.

**Figure 4:**
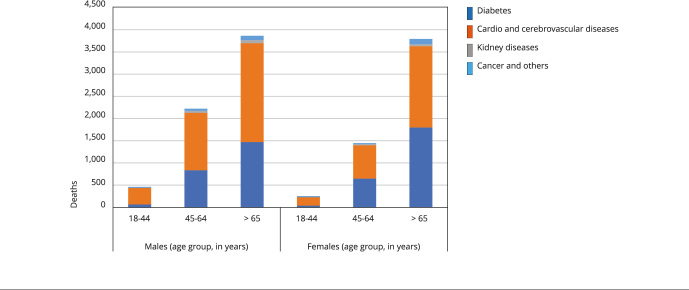
Sugar-sweetened beverages attributable deaths in adults.

It was further estimated that a total of 139,623 cardiovascular events, 93,133 strokes, 7,943 cases of musculoskeletal disease (mainly low back pain), 74,725 cases of chronic kidney disease, and 27,333 cases of asthma are attributable to SSBs consumption. Figure 4 shows the relative burden of mortality related to SSBs consumption, showing the high proportion of deaths attributable to type 2 diabetes mellitus, CVD and cerebrovascular diseases. Moreover, 13,020 deaths were found, corresponding to 2.98% of deaths from these causes in Brazil. These values represent 362,088 DALYs and an annual cost of USD 3,050 million per year.

### Sensitivity analysis

The findings from the sensitivity analysis are presented in Table 3 as values that compose the 95%CI around the central value of the number of events, deaths, and costs for each condition and sex.

## Discussion

This model estimated that approximately 13,000 deaths, more than 1,800,000 cases of disease events, 2,219,168 cases of overweight or obesity in the adult population, 722,639 cases of overweight or obesity in children and adolescents, and USD 3,050 million per year spent in direct medical costs could be attributable to SSBs consumption in Brazil.

Our results are consistent with previous studies for the Americas region in both the direction and magnitude of the effects of SSBs on health and economic outcomes. A study by Singh et al. ^10^, which considered only the effects of SSBs consumption mediated by BMI, have reported a substantial absolute and relative burden of mortality and morbidity related to SSBs consumption in Latin America in 2010. In the case of Brazil, it was reported that SSBs are responsible for 13,733 deaths (including deaths from CVD, diabetes mellitus, and different types of cancer), which represents 2.3% of deaths from these types of diseases ^10^. Similarly, Singh et al. ^10^ calculated that SSBs are responsible for approximately 8.5 million DALYs globally, of which 195,424 DALYs (95%CI: 111,520-315,353) correspond to Brazil, a value inferior to our estimation. One of the probable explanations for this difference is that this model includes more pathologies that generate a high burden in years of life with disability, such as musculoskeletal problems ^10^.

Although some studies reported a slight decrease in the consumption of SSBs in recent years, consumption is still high, and a significant portion of the Brazilian population consume SSBs daily ^23^. Some of the factors that appear to increase the SSBs consumption are exposure to advertisements and other forms of publicity, the availability of SSBs in schools and restaurants, the relatively low prices of these products, distrust in the safety of tap water, consumption patterns, and ignorance or disbelief of the association between SSBs consumption with weight gain and other diseases ^3^ ^,^ ^24^.

The results of our model to assess the burden of diseases attributable to SSBs consumption reveals the need to strengthen policies to reduce their consumption. Measures to reduce SSBs consumption are a political axis for the prevention of NCDs across the region ^24^ ^,^ ^25^. Evidence has pointed that increasing taxes on these products can bring public benefits, with Mexico being the first country to successfully promote this type of change in the region ^26^. In the case of Brazil, Claro et al. ^27^ have reported that a 1% increase in the price of SSBs can lead to a 0.85% reduction in calories consumed from these products (1.03% reduction for the population below the poverty line and 0.63% for the population above it).

Another possible policy to be implemented, the reformulation of SSBs to reduce sugar content, could also produce significant reductions in consumption among the populations of the Americas ^28^. Likewise, warning labels could have a beneficial effect on public health and obesity and its associated costs. The expected impact of beverage labeling was estimated at a 10.5% reduction in calories consumed by these ultra-processed products ^29^. Mexico, Chile, Peru, Uruguay, and recently Argentina have already implemented the labeling of ultra-processed foods and are beginning to benefit from these changes.

Low- and middle-income countries and Latin America suffer a disproportionate disease burden and death attributable to SSBs consumption ^10^. However, it is a region where health policy-driven interventions could be beneficial since many countries present low price of SSBs, lack of advertising regulation and/or suboptimal implementation of existing regulations, lack of public awareness to the risks associated with SSBs, and absence of warning labels on foods. This study can be a valuable contribution to raising awareness among the population and decision-makers about this important public health problem in Brazil and support policy interventions that many countries are struggling to implement.

This model was developed based on epidemiological and cost information for Brazil. It included a comprehensive review of the best available evidence, along with the effects of SSBs on weight, type 2 diabetes mellitus, and cardiovascular disease. The additional inclusion of recent evidence on the direct effect of SSBs on both diabetes mellitus and CVD (independent of BMI) has contributed to results that more accurately reflect the epidemiological reality of the country and region ^18^ ^,^ ^19^. Finally, the early involvement of key decision-makers in this study provides the information prioritized by experts in the field to drive policy change.

One of the main limitations of our study is that the results rely heavily on information on epidemiological parameters, SSBs consumption, and costs. Thus, the data quality and availability may be limited in many countries of the region. Our analysis considered the essential set of health outcomes related to SSBs. However, it may represent an underestimate of the actual burden since other conditions, such as dental decay and social and psychological impact of suffering from obesity, were not included in our model. Moreover, cost estimate of illnesses linked to SSBs consumption may be underestimated since we used data from the public sub-sector. Furthermore, only the direct medical costs generated by SSBs were considered, which are only a part of the financial burden to society since other social costs exist, such as productivity loss, school absenteeism, and informal caregiver time cost ^30^. Lastly, in our study, the impact of SSBs consumption was modeled by a hypothetical exercise of no consumption, without replacing it for other sugary products. Other modeling exercises have incorporated the consumption shift to other products, so the benefits of reduced consumption of SSBs may be diminished.

## Conclusion

This study estimated the burden of diseases attributable to SSBs consumption and the associated direct medical costs in Brazil.
